# Curcumin Ameliorates Palmitic Acid-Induced Saos-2 Cell Apoptosis Via Inhibiting Oxidative Stress and Autophagy

**DOI:** 10.1155/2021/5563660

**Published:** 2021-03-26

**Authors:** Baicheng Ma, Gaopeng Guan, Qizhuang Lv, Lei Yang

**Affiliations:** ^1^Jiangxi Provincial Key Lab of System Biomedicine, Jiujiang University, Jiujiang 332000, Jiangxi, China; ^2^School of Medicine, Jiujiang University, Jiujiang 332000, Jiangxi, China; ^3^Affiliated Hospital of Jiujiang University, Jiujiang University, Jiujiang 332000, Jiangxi, China; ^4^College of Biology & Pharmacy, Yulin Normal University, Yulin 537000, Guangxi, China

## Abstract

**Objectives:**

We aimed to determine the effects of curcumin on palmitic acid- (PA-) induced human osteoblast-like Saos-2 cell apoptosis and to explore the potential molecular mechanisms in vitro level.

**Methods:**

Saos-2 cell were cultured with PA with or without curcumin, N-acetylcysteine (NAC, anti-oxidant), 3-methyladenine (3-MA, autophagy inhibitor) AY-22989 (autophagy agonist) or H_2_O_2_. Then, the effects of PA alone or combined with curcumin on viability, apoptosis, oxidative stress, and autophagy in were detected by CCK-8, flow cytometry assay and western blot.

**Results:**

We found that autophagy was induced, oxidative stress was activated, and apoptosis was promoted in PA-induced Saos-2 cells. Curcumin inhibited PA-induced oxidative stress, autophagy, and apoptosis in Saos-2 cells. NAC successfully attenuated oxidative stress and apoptosis, and 3-MA attenuated oxidative stress and apoptosis in palmitate-induced Saos-2 cells. Interestingly, NAC inhibited PA-induced autophagy, but 3-MA had no obvious effects on oxidative stress in PA-treated Saos-2 cells. In addition, curcumin inhibited H_2_O_2_ (oxidative stress agonist)-induced oxidative stress, autophagy, and apoptosis, but curcumin had no obvious effect on AY-22989 (autophagy agonist)-induced autophagy and apoptosis.

**Conclusion:**

The present study demonstrated that oxidative stress is an inducer of autophagy and that curcumin can attenuate excess autophagy and cell apoptosis by inhibiting oxidative stress in PA-induced Saos-2 cells.

## 1. Introduction

Diabetes mellitus is a pandemic metabolic disease and has a worldwide distribution. Patients with diabetes mellitus have various skeletal disorders, including osteopenia or osteoporosis [1]. Diets rich in high-fat foods, especially saturated fats, are usually the cause of the clinical symptoms of metabolic syndrome, such as obesity, insulin resistance, and type 2 diabetes, which eventually increase the likelihood of osteoporosis [2]. Moreover, obesity and type 2 diabetes trigger a prolonged elevation of circulating free fatty acid levels (FFAs) especially the saturated FFAs such as palmitate (PA), which causes lipotoxicity in many cell types, including human osteoblast-like Saos-2 cell [3]. Additionally, PA-induced lipotoxicity plays a vital role in the development and progression of osteoporosis [4, 5]. Numerous studies have focused on factors involved in the mechanism of PA-induced lipotoxicity, such as oxidative stress and autophagy [5].

Oxidative stress is essentially an imbalance between the generation of reactive oxygen species (ROS) and the ability of the body to counteract or detoxify their harmful effects through neutralization by antioxidants [6]. ROS are produced in all cellular compartments as a result of exposure to toxic agents and natural by-products of mitochondrial respiration and can disrupt the normal mechanisms of cellular signaling and function, resulting in DNA damage and apoptosis [7]. Previous studies have reported that oxidative stress plays an important role in the pathophysiology of many diseases, including osteoporosis [6].

Autophagy is a complex catabolic process in eukaryotes that enables cells to recycle cytoplasmic components through degradation in the lysosomes. Under stressful conditions, such as nutrient deprivation and oxidative stress, autophagy is activated as a pathway to promote cell survival by maintaining energy and reducing toxic substances [8]. In addition, there is increasing evidence that excessive or uncontrolled levels of autophagy may be essential for cell apoptosis in certain settings [9]. Moreover, some studies have reported that autophagy is related to diabetic osteoporosis, [8] and oxidative stress has been reported to be a novel autophagy inducer [10].

Curcumin, a non-ﬂavonoid polyphenol found in the plant Curcuma longa, has been extensively investigated because of its anti-inflammatory, anti-oxidative, and cytoprotective properties [11]. Previous studies have reported that curcumin is a promising drug for the prevention and treatment of diabetes and diabetes-related diseases, including osteoporosis [12]. Moreover, both oxidative stress and autophagy are related to diabetic osteoporosis [13]. In addition, previous study has reported that curcumin can regulate oxidative stress and autophagy in vivo and in vitro [14].

In this study, we aimed to determine the effects of curcumin on PA-induced human osteoblast-like Saos-2 cell apoptosis and to explore the potential molecular mechanisms in vitro level. Herein, we investigated the participation and relationship of oxidative stress and autophagy and evaluated the effects and molecular mechanisms of curcumin in PA-induced Saos-2 cell apoptosis.

## 2. Materials and Methods

### 2.1. Cell Culture and Treatment

Saos-2 cells were cultured in DMEM supplemented with 10% FBS, 50 *μ*g/mL penicillin, and 50 *μ*g/mL gentamicin in a humidified incubator at 37°C with 5% CO_2_. When the cells reached 70%–80% confluence, they were treated with different concentrations (1.25–20 *μ*M) of curcumin and 200 *µ*M PA to determine their effects on cell activity. At the same time, the cells were exposed to 200 *µ*M PA in the presence or absence of 10 *µ*M curcumin, 5 mM 3-MA, 10 *µ*M AY-22989, 100 *µ*M H_2_O_2_, or 2 mM NAC for 24 h.

### 2.2. Measurement of Cell Viability

After the treatment, 10 *μ*l of CCK-8 was added to each well, and the cells were incubated for 2 h at 37°C. The number of viable cells was measured at 450 nm with a microplate reader (Bio-Rad 680). The results are presented as a percentage of the values measured for untreated control cells.

### 2.3. Cell Apoptosis Measurement

After the treatment, cell apoptosis was quantified with an Annexin V-FITC/PI Apoptosis Analysis Kit according to the manufacturer's instructions. The cells were analyzed using a flow cytometer (Becton, Dickinson and Company, USA) within 1 h. Early apoptotic cells were determined by counting the percentage of Annexin V-FITC+/PI− cells, progressed apoptotic cells were obtained by counting the percentage of Annexin V-FITC+/PI+ cells, and necrotic cells were detected by counting the percentage of Annexin V-FITC−/PI+ cells and Annexin V-FITC−/PI− cells.

### 2.4. Western Blot

After the treatment, cells were collected and washed with ice-cold PBS and lysed with RIPA buffer, and the total protein concentration was measured using the BCA assay. Fifty micrograms of total protein from each sample were loaded into each well of a 13% SDS-PAGE gel, and then, proteins were separated by electrophoresis. Proteins were then transferred to PVDF membranes. After blocking in TBST with 10% nonfat milk for 2 h, the samples were incubated with primary antibody against B-cell lymphoma-2 (Bcl-2, 1 : 1,000), Bcl-2 associated X protein (BAX, 1 : 500), glyceraldehyde-phosphate dehydrogenase (GAPDH, 1 : 2,000), Autophagy protein 5 (ATG5, 1 : 1,000), polyubiquitin-binding protein (P62, 1 : 1,000), and microtubule-associated protein light chain 3 (LC3, 1 : 1,000) overnight at 4°C. After washing, the membranes were incubated with secondary antibody (1 : 4,000) conjugated to horseradish peroxidase at 37°C for 30 min. The immunoreactive bands were visualized using a Super Signal West Pico kit according to the manufacturer's instructions, and the densities of the protein bands were measured by densitometric analysis using ImageJ software 1.48 (National Institutes of Health, Bethesda, Maryland, USA).

### 2.5. mCherry-GFP-LC3 Adenovirus Transfection and Autophagy Assay

Cells cultured in 24-well plates (1 × 105 cells/well) were transfected with mCherry-GFP-LC3 adenovirus at 40 MOI for 24 h. After transfection, the cells were incubated with fresh culture medium again for 24 h. The numbers of GFP and mCherry dots per cell were counted in three randomly selected fields under a fluorescence microscope (Olympus Corporation, Tokyo, Japan). GFP degrades in an acidic environment, while mCherry does not. Thus, yellow spots (formed from the overlap of red and green) indicate autophagosomes, while red spots indicate autophagic lysosomes. If autophagy is activated, the red signal will dominate over yellow. If autophagy is suppressed, there will be more yellow signal than red signal.

### 2.6. ROS Measurements

After the treatment, the cells were incubated with DCFH-DA (10 *µ*M) at 37°C for 20 min. Then, the cells were subsequently washed three times with PBS, and the ROS-sensitive dye in cells was assessed under an inverted fluorescence microscope (Olympus Corporation, Tokyo, Japan) with an excitation wavelength of 488 nm and an emission wavelength of 525 nm. Fluorescent intensity was acquired using ImageJ 1.48 image analysis software.

### 2.7. Superoxide Dismutase (SOD) Detection

After the treatment, the cells were harvested and sonicated in PBS containing 1.0 mM PMSF to obtain cell homogenates. The homogenates were then centrifuged at 15,000 ×*g* and 4°C for 15 min, and the protein content was determined using the BCA Protein Assay Kit. Then, the supernatants were used for measuring cellular SOD. The SOD activity was determined at 450 nm using a microplate reader (Bio-Rad 680).

### 2.8. Caspase-3 Activity Measurement

After the treatment, the cells were harvested by centrifugation and incubated in lysis buffer on ice for 15 min. The lysates were then centrifuged at 15,000 ×*g* and 4°C for 15 min, and the protein content was determined using the BCA Protein Assay Kit according to the manufacturer's instructions. Then, each sample was incubated with the Caspase-3 substrate at 37°C in a microplate for 4 h. The samples were measured at 405 nm using a microplate reader.

### 2.9. Statistical Analyses

All experiments were repeated at least three times for each group, and the data are expressed as the mean ± standard error of the mean (SEM). The data were analyzed by one-way analysis of variance (ANOVA) followed by Fisher's least significant difference test and independent samples Student's *t*-test with SPSS software, version 13.0 (SPSS, Chicago, IL, USA). Differences were considered statistically significant when *P* < 0.05.

## 3. Results

### 3.1. Curcumin Attenuated the PA-Induced Cell Viability Decrease and Cell Apoptosis in Saos-2 Cells

**Figure 1:**
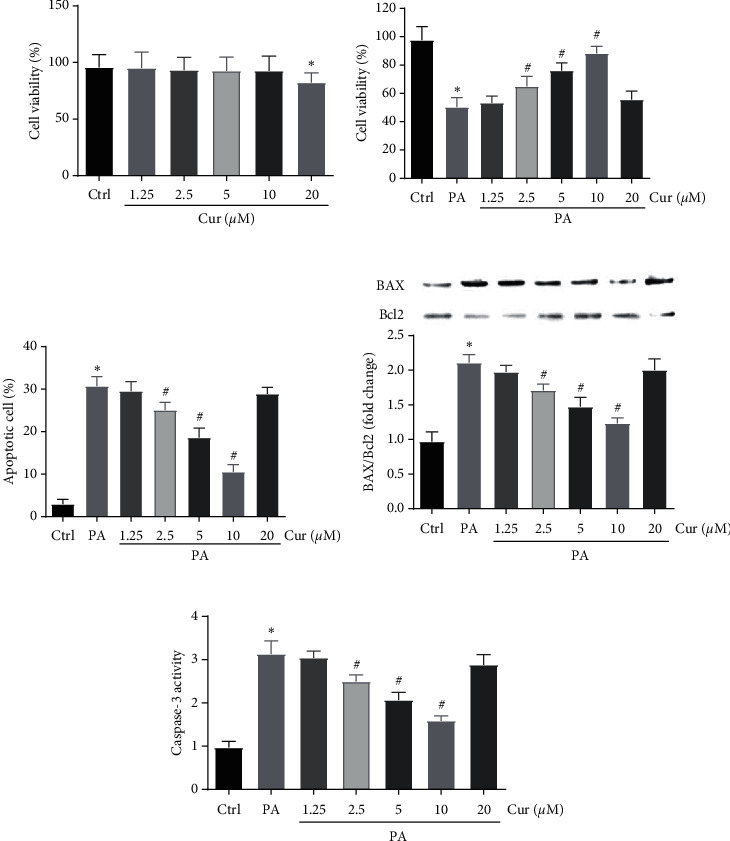
Effects of curcumin on PA-induced cell viability and apoptosis in Saos-2 cells. Saos-2 cells were treated with different concentrations of curcumin for 24 h. Cell viability was measured using CCK-8 assay (a). Cells were incubated with 200 *μ*M PA for 24 h with or without curcumin at different concentrations. Cell viability was measured using CCK-8 assay (b). Apoptosis analysis was performed via flow cytometry (c). The protein expression levels of Bcl-2 and BAX were detected by western blot analysis. The analysis of band intensities is presented as the relative ratio of BAX to Bcl-2 (d). The Caspase-3 activity was detected using a microplate reader (e). Ctrl, control; Cur, different concentrations of curcumin; PA, 200 mM palmitic acid. Statistical analysis is shown on the bar graphs. Data are presented as the mean ± SEM of the three independent experiments. ^*∗*^*P* < 0.05 versus control; ^#^*P* < 0.05 versus PA.

We first assessed the cytotoxicity of curcumin (1.25, 2.5, 5, 10, 20 *μ*M) on Saos-2 cells. The CCK-8 results showed that 1.25–10 *μ*M curcumin had no significant cytotoxicity, but 20 *μ*M curcumin decreased cell viability after 24 h of culture (Figure 1(a)). Moreover, 200 *µ*M PA and 1.25–20 *μ*M curcumin were added to the culture medium for 24 h to determine the effects of curcumin on PA-induced Saos-2 cell viability. The results showed that 200 *µ*M PA reduced cell viability by about 50%, and 1.25–10 *μ*M curcumin prevented this reduction in cell viability in a dose-dependent manner. However, 20 *μ*M curcumin had no obvious effects on cell viability compared with the control treatment (Figure 1(b)). Flow cytometry analysis revealed that curcumin prevented cell apoptosis caused by PA in a dose-dependent manner. However, 20 *μ*M curcumin had no obvious effects on PA-induced cell apoptosis (Figure 1(c)). The colorimetric assay and western blot results showed that curcumin attenuated the expression of BAX and Caspase-3 but increased the expression of Bcl-2 in PA-treated Saos-2 cells (Figures 1(d) and 1(e)).

### 3.2. Curcumin Reduced PA-Induced Oxidative Stress and Autophagy in Saos-2 Cells

**Figure 2:**
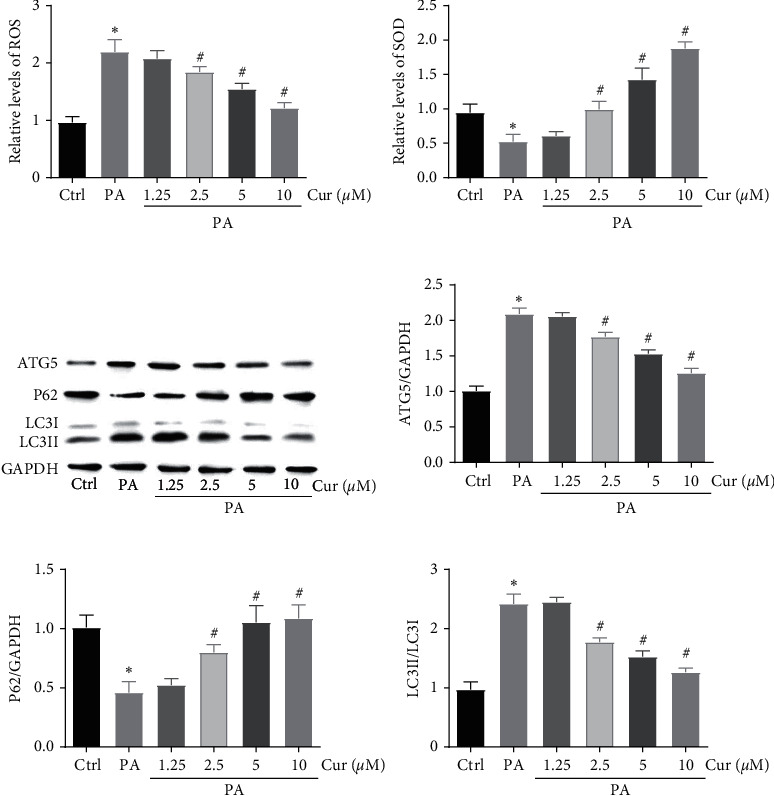
Effects of curcumin on PA-induced oxidative stress and autophagy in Saos-2 cells. Saos-2 cells were incubated with 200 *μ*M PA for 24 h with or without curcumin at different concentrations. The generation of ROS was measured by using the DCFH-DA. Saos-2 cells were stained with DCFH-DA and analyzed by fluorescent microscopy. Statistical analysis of fluorescence intensity in Saos-2 cells by image J software (a). The relative levels of SOD were detected by a microplate reader (b). Western blot detected ATG5, P62, and LC3 (c). Ratio of ATG5 and P62 to GAPDH, and ratio of LC3- II to LC3- I (d, e, and f). Ctrl, control; Cur, different concentrations of curcumin; PA, 200 mM palmitic acid. Statistical analysis is shown on the bar graphs. Data are presented as the mean ± SEM of the three independent experiments. ^*∗*^*P* < 0.05 versus control; ^#^*P* < 0.05 versus PA.

To determine the effects of curcumin on oxidative stress and autophagy in PA-treated Saos-2 cells, the cells were treated with 200 *µ*M PA with or without 1.25–10 *μ*M curcumin for 24 h, and oxidative stress-related parameters were analyzed. DCFH-DA assay revealed that PA increased intracellular ROS production, but curcumin treatment decreased the PA-induced ROS generation in a dose-depend manner (Figure 2(a)). Moreover, PA reduced the activity of the anti-oxidant enzyme SOD, which was rescued by 1.25–10 *μ*M curcumin in a dose-dependent manner (Figure 2(b)). These results showed that curcumin attenuates PA-induced cell apoptosis in Saos-2 cells. The western blot results indicated that the 200 *μ*M PA treatment increased the protein expression of ATG5 and LC3II and the degradation of p62. Meanwhile, we found that the protein expression levels of ATG5 and LC3II in PA-treated cells significantly decreased but that of p62 increased following the addition of different doses of curcumin (Figures 2(c)–2(f)).

### 3.3. Roles of Oxidative Stress and Autophagy in the Protective Effect of Curcumin on PA-Induced Saos-2 Cell Apoptosis

**Figure 3:**
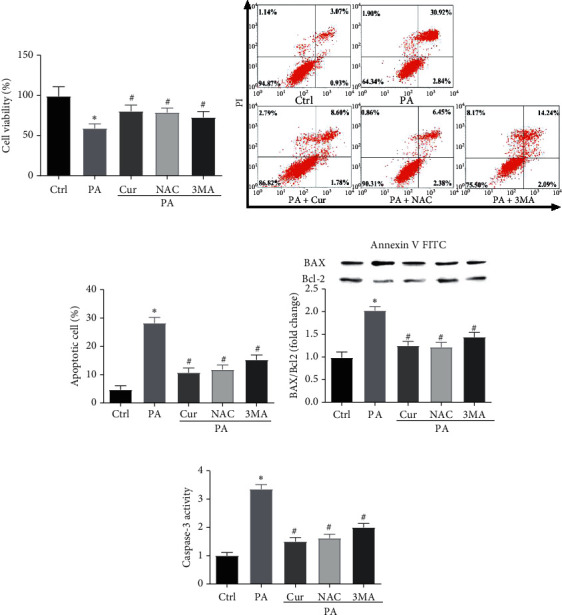
Oxidative stress and autophagy is involved in curcumin against PA-induced Saos-2 cell apoptosis (a). Saos-2 cells were incubated with 200 *μ*M PA for 24 h with or without curcumin, NAC, or 3-MA. Cell viability was measured using CCK-8 assay (b). Apoptosis analysis was performed via flow cytometry (c). The protein expression levels of Bcl-2 and BAX were detected by western blot analysis. The analysis of band intensities is presented as the relative ratio of BAX to Bcl-2 (d). The Caspase-3 activity was detected using a microplate reader (e). Ctrl, control; Cur, 10 *µ*M curcumin; PA, 200 mM palmitic acid; NAC, 2 mM N-acetylcysteine; 3-MA/3-MA, 5 mM 3-methyladenine. Statistical analysis is shown on the bar graphs. Data are presented as the mean ± SEM of the three independent experiments. ^*∗*^*P* < 0.05 versus control; ^#^*P* < 0.05 versus PA.

Because both oxidative stress and autophagy can be induced during Saos-2 cell apoptosis, we designed the following experiment to determine whether oxidative stress and autophagy are involved in PA-induced Saos-2 cell apoptosis. The oxidative stress inhibitor NAC and autophagy inhibitor 3-MA were added to the culture medium during the PA treatment. The CCK-8 assay results showed that NAC and 3-MA clearly increased cell viability in PA-induced Saos-2 cells (Figure 3(a)). The flow cytometry results indicated that NAC and 3-MA reduced cell apoptosis caused by PA (Figures 3(b) and 3(c)). The western blot and colorimetric assay results demonstrated that the decreases in BAX and Caspase-3 expression and the increase in Bcl-2 expression were also rescued by NAC and 3-MA in PA-induced Saos-2 cells (Figures 3(d) and 3(e)). Moreover, NAC and 3-MA had the same protective effect as curcumin on PA-induced Saos-2 cell apoptosis. Therefore, these results indicate that PA induces Saos-2 cell apoptosis by stimulating oxidative stress and activating autophagy and that curcumin may attenuate cell apoptosis by inhibiting oxidative stress and autophagy.

### 3.4. Relation of Oxidative Stress and Autophagy to the Protective Effect of Curcumin on PA-Induced Saos-2 Cell

**Figure 4:**
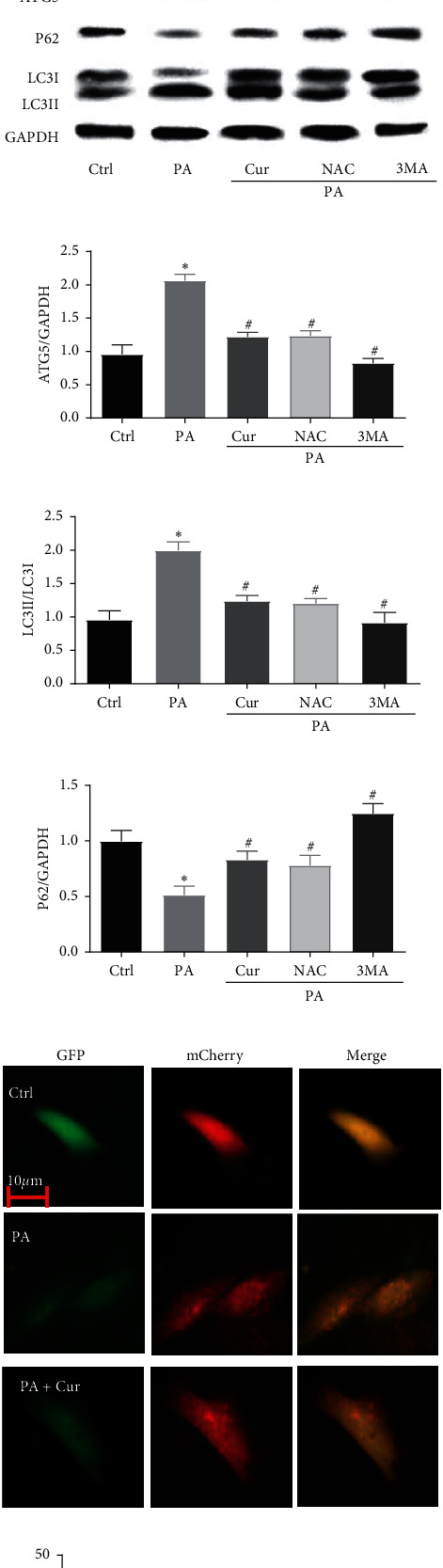
Curcumin reduces oxidative stress and oxidative stress-induced autophagy in PA-induced Saos-2 cell. Saos-2 cells were incubated with 200 *μ*M PA for 24 h with or without curcumin, NAC, or 3-MA. The generation of ROS was measured by using the DCFH-DA. Saos-2 cells were stained with DCFH-DA and analyzed by fluorescent microscopy (a). Statistical analysis of fluorescence intensity in Saos-2 cells by image J software (b). The relative levels of SOD were detected by a microplate reader (c). Western blot detected ATG5, P62, LC3I, and LC3II (d). Ratio of ATG5 and P62 to GAPDH, and ratio of LC3- II to LC3- I (e, f, and g). Fluorescent microscopy analysis of Saos-2 cells transfected with mCherry-GFP-LC3B adenovirus (h). Statistical analysis of fluorescent dots in Saos-2 cells (i). Yellow spots indicate autophagosomes, and red spots indicate autolysosomes after the pictures were merged. Ctrl, control; Cur, 10 *µ*M curcumin; PA, 200 mM palmitic acid; NAC, 2 mM N-acetylcysteine; 3-MA/3-MA, 5 mM 3-methyladenine. Statistical analysis is shown on the bar graphs. Data are presented as the mean ± SEM of the three independent experiments. ^*∗*^*P* < 0.05 versus control; ^#^*P* < 0.05 versus PA.

To determine whether there is an association between oxidative stress and autophagy in PA-induced Saos-2 cells, we assessed the oxidative stress parameters and autophagy makers in PA-induced Saos-2 cells with or without NAC and 3-MA. The oxidative stress parameter results indicated that NAC, but not 3-MA, inhibited the ROS generation and SOD downregulation (Figures 4(a)–4(c)), suggesting that NAC, but not 3-MA, abrogated PA-induced oxidative stress. In addition, NAC and 3-MA clearly reduced the protein levels of ATG5 and LC3II and the degradation of P62 (Figures 4(d)–4(g)). Moreover, the NAC and 3-MA treatments inhibited the PA-induced increases in autophagosomes and autolysosomes (Figures 4(h) and 4(i)), suggesting that NAC and 3-MA alleviated PA-activated autophagy. Therefore, NAC, but not 3-MA, had the same effect as curcumin on PA-induced oxidative stress and autophagy in Saos-2 cells. These results indicated that oxidative stress is an inducer of autophagy in PA-induced Saos-2 cells and that curcumin may reduce oxidative stress and oxidative stress-induced autophagy in PA-induced Saos-2 cells.

### 3.5. Effects of Curcumin on Oxidative Stress- or Autophagy-Induced Saos-2 Cell Apoptosis

**Figure 5:**
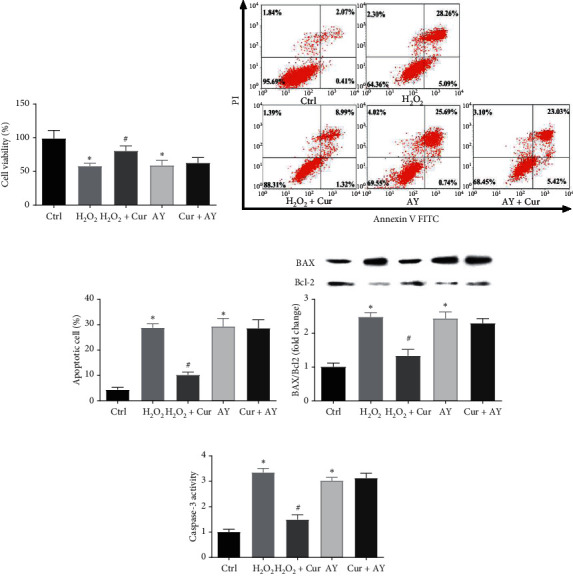
Curcumin attenuated oxidative stress-induced Saos-2 cell apoptosis. Saos-2 cells were incubated with H_2_O_2_ or AY-22989 for 24 h with or without curcumin. Cell viability was measured using CCK-8 assay (a). Apoptosis analysis was performed via flow cytometry using Annexin V-FITC/PI double staining (b, c). The protein expression levels of Bcl-2 and BAX were detected by western blot analysis. The analysis of band intensities is presented as the relative ratio of BAX to Bcl-2 (d). The Caspase-3 activity was detected using a microplate reader (e). Ctrl: control; Cur: 10 *µ*M curcumin; H_2_O_2_: 100 *µ*M H_2_O_2_; AY: 10 *µ*M AY-22989. Statistical analysis is shown on the bar graphs. Data are presented as the mean ± SEM of the three independent experiments. ^*∗*^*P* < 0.05 versus control, ^#^*P* < 0.05 versus PA.

To further explored the roles of oxidative stress and autophagy in the mechanism of the protective effect of curcumin on PA-induced Saos-2 cell apoptosis, we added the oxidative stress inducer H_2_O_2_ or the autophagy agonist AY-22989 to the culture medium with or without curcumin. The results showed that H_2_O_2_ and AY-22989 clearly decreased cell viability and induced cell apoptosis in Saos-2 cells (Figures 5(a)–5(c)). Curcumin improved cell viability, prevented cell apoptosis, and altered the expression of apoptotic regulatory genes (Caspase-3, BAX, and Bcl-2) in H_2_O_2_-treated Saos-2 cells (Figures 5(d) and 5(e)). However, curcumin did not have a protective effect on AY-22989-induced Saos-2 cell apoptosis and cell viability decrease. These results indicated that curcumin inhibited oxidative stress-induced apoptosis not autophagy-induced apoptosis in Saos-2 cells.

### 3.6. Effects of Curcumin on Oxidative Stress or Autophagy in Saos-2 Cells

**Figure 6:**
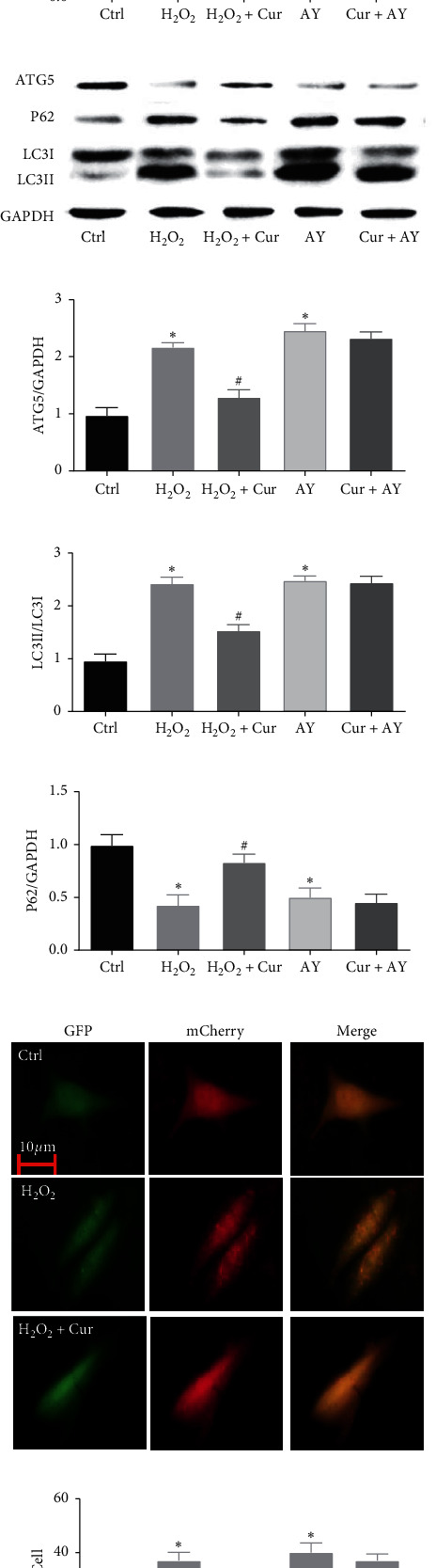
Curcumin inhibited oxidative stress and autophagy in H_2_O_2_- induced Saos-2 cells apoptosis. Saos-2 cells were incubated with H_2_O_2_ or AY-22989 for 24 h with or without curcumin. The generation of ROS was measured by using the DCFH-DA. Saos-2 cells were stained with DCFH-DA and analyzed by Fluorescent microscopy (a). Statistical analysis of fluorescence intensity in Saos-2 cells by image J software (b). The relative levels of SOD were detected by a microplate reader (c). Western blot detected ATG5, P62, LC3I and LCII (d). Ratio of ATG5 and P62 to GAPDH, and ratio of LC3- II to LC3- I (e) (f), and (g). Fluorescent microscopy analysis of Saos-2 cells transfected with mCherry-GFP-LC3B adenovirus (h). Statistical analysis of fluorescent dots in Saos-2 cells (i). Yellow spots indicate autophagosomes, and red spots indicate autolysosomes after the pictures were merged. Ctrl: control; Cur: 10 *µ*M curcumin; H_2_O_2_: 100 *µ*M H_2_O_2_; AY: 10 *µ*M AY-22989. Statistical analysis is shown on the bar graphs. Data are presented as the mean ± SEM of the three independent experiments. ^*∗*^*P* < 0.05 versus control, ^#^*P* < 0.05 versus PA.

Moreover, we assessed oxidative stress and autophagy after adding H_2_O_2_ or AY-22989 to the culture medium with or without curcumin. The results showed that H_2_O_2_ increased the levels of ROS and inhibited the activity of SOD in Saos-2 cells (Figures 6(a)–6(c)). Curcumin reduced the levels of ROS and increased the activity of SOD in H_2_O_2_-treated Saos-2 cells. AY-22989 did not change the levels of ROS or the activity of SOD in Saos-2 cells. The western blot analysis showed that the expression of ATG5 and LC3II and the degradation of P62 increased in H_2_O_2_- and AY-22989-treated Saos-2 cells (Figures 6(d)–6(g)). We also found that the formation of autophagosomes and autolysosomes increased, suggesting that H_2_O_2_ and AY-22989 activated autophagy in Saos-2 cells (Figures 6(h) and 6(i)). Curcumin reduced the expression of ATG5 and LC3II, degradation of P62, and formation of autophagosomes and autolysosomes in H_2_O_2_-treated but not AY-22989-treated Saos-2 cells. These results indicated that curcumin inhibited oxidative stress and oxidative stress-induced autophagy but did not have a direct effect on autophagy in Saos-2 cells.

## 4. Discussion

**Figure 7:**
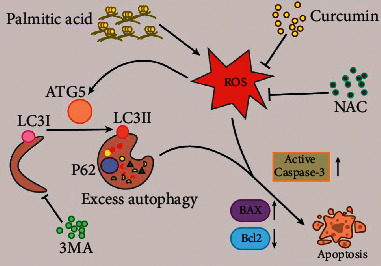
The mechanism by which curcumin ameliorates palmitic acid-induced Saos-2 cell apoptosis.

In the past few decades, with the dramatic increase in the type 2 diabetes epidemic, our understanding of the role of dyslipidemia and lipotoxicity in many diseases has grown considerably. The association between hyperlipidemia and osteoporosis has been established clinically and in experimental models [15]. Palmitate is the main saturated FFA in plasma that stimulates ROS production and autophagy activity in cultured cardiomyocytes and endothelial cells [16]. In our previous study, we reported that PA triggers Saos-2 cell apoptosis via excessive autophagy [3], and several studies have reported that curcumin protects cells against oxidative stress-mediated apoptosis [14, 17]. However, the cryoprotective effect of curcumin on PA-treated cells and the underlying mechanisms have not been reported. Here, we demonstrated for the first time that curcumin can attenuate PA-induced Saos-2 cell apoptosis via inhibiting oxidative stress and autophagy (Figure 7).

Curcumin has a range of pharmacological effects, including anticancer, anti-inflammatory, antioxidative as well as antioxidant, and cryoprotective activities [18, 19]. In our research, curcumin rescued the PA-induced cell viability decrease and cell apoptosis. Our results are different from those of previous studies that reported that curcumin triggers apoptosis in many tumor cells, including HT29 cells [20] and upper aerodigestive tract cancer cells [21], suggesting that curcumin is a potential anticancer drug. Meanwhile, our results further supported the conclusion that curcumin protects cells against various injuries such as ischemic injury [18, 22] and diabetes [23, 24] in vivo and in vitro. Therefore, curcumin seems to have a bidirectional effect on cell proliferation and apoptosis. On the one hand, it can promote cell apoptosis, especially in cancer cells; on the other hand, it can prevent cell apoptosis. The reasons for the different results (cell die or live) caused by curcumin may be due to differences in cell types, drug doses, and experimental methods.

We further examined apoptotic regulatory genes, including Caspase-3, BAX, and Bcl-2. The results showed that curcumin decreased the expression of the pro-apoptotic molecule BAX and increased the expression of the anti-apoptotic molecule Bcl-2. Our results were consistent with the findings of previous studies that reported that curcumin ameliorated cell apoptosis by regulating Bcl-2 family proteins, which can downregulate BAX and upregulate Bcl-2 expression [25]. Caspase-3 acts as an executioner in Caspase-mediated apoptosis, and the expression of Caspase-3 positively correlates with the rate of apoptosis in cells [11]. In our study, we found that curcumin can inhibit Caspase-3 activity, suggesting that curcumin can maintain cell survival by restraining Caspase-3. Therefore, we propose that curcumin affects cell proliferation and apoptosis in PA-treated cells by regulating Bcl-2 family proteins and mitigating Caspase-3 activity.

In previous studies, oxidative stress was found to be involved in the process of PA-mediated apoptosis [7, 26]. In addition, ROS production is a particularly destructive aspect of oxidative stress. Apoptosis is induced by excess free radicals when ROS production exceeds the capacity of antioxidant defenses [7]. In our study, we found that PA enhanced ROS generation. SOD is an antioxidant that protect cells against oxidative damage. However, the downregulation of SOD further promotes oxidative stress and damages cells. Previous studies have demonstrated that curcumin can prevent cell damage via suppressing oxidative stress [27]. Our data indicated that curcumin alleviates oxidative stress in a dose-dependent manner, indicating the protective ability of curcumin to prevent PA-induced oxidative stress in Saos-2 cells.

Autophagy is an important intracellular bulk degradation process involving the lysosome-dependent turnover of damaged cytosolic proteins and organelles, and it is critical for the maintenance of the normal cell phenotype and its functions. However, excess autophagy triggers cell apoptosis by destroying large proportions of the cytosol and organelles [28]. In our previous study we found that PA activated autophagy in a dose- and time-dependent manner in Saos-2 cells. Moreover, the PA-induced excessive autophagy caused cell apoptosis in Saos-2 cells. Furthermore, we analyzed the effects of curcumin on PA-induced autophagy. The results showed that PA successfully induced the expression of autophagy-related genes and that curcumin inhibited autophagy in a dose-dependent manner, suggesting that curcumin blocked.

To clarify whether oxidative stress and autophagy triggered PA-induced Saos-2 cell apoptosis, we used the oxidative stress inhibitor NAC and the autophagy inhibitor 3-MA to treat Saos-2 cells during the PA treatment. Apoptosis was inhibited by NAC and 3-MA. These results further indicated that PA-induced oxidative stress and autophagy cause Saos-2 cell apoptosis. Together, the results that curcumin inhibits autophagy, oxidative stress, and apoptosis indicate that curcumin may attenuate cell apoptosis by inhibiting oxidative stress and autophagy in PA-induced Saos-2 cells.

Previous studies have shown that oxidative stress can induce autophagy under starvation conditions [29]. Consistent with this previous study, we found that H_2_O_2_ activated autophagy in Saos-2 cells. Moreover, the PA-induced autophagy activity was inhibited by NAC in Saos-2 cells. In addition, AY-22989 and 3-MA did not have an effect on Saos-2 cells with or without PA treatment. These results indicated that oxidative stress is an inducer of autophagy in PA-induced Saos-2 cells. However, it is still unclear whether curcumin directly affects autophagy in PA-induced Saos-2 cells. Moreover, some studies have also reported that curcumin induces autophagy activation, such as in osteosarcoma MG63 cells [30] and oral cancer cells [31].

To further determine the effects of curcumin on autophagy in Saos-2 cells, we used AY-22989 to activate autophagy during the treatment. The results showed that curcumin had no obvious effects on autophagy and autophagy-induced apoptosis, indicating that curcumin has no effect on autophagy in Saos-2 cells. In addition, we found that the apoptosis, oxidative stress, and autophagy induced by H_2_O_2_ were rescued by curcumin, suggesting that curcumin can inhibit oxidative stress-induced autophagy and apoptosis in Saos-2 cells.

In summary, we found that curcumin can inhibit PA-induced oxidative stress, autophagy, and apoptosis. Moreover, previous studies have reported that oxidative stress can activate these processes in many cells and tissues [7, 32]. Our results showed that NAC can inhibit PA-induced autophagy but that 3-MA had no obvious effects on oxidative stress in Saos-2 cells with PA treatment. Thus, we assume that curcumin indirectly inhibits the PA-induced autophagy via suppressing oxidative stress. To further clarify this hypothesis, we found that curcumin inhibited H_2_O_2_-induced oxidative stress, autophagy, and apoptosis, but curcumin had no obvious effects on AY-22989-induced autophagy. Therefore, curcumin ameliorates PA-induced human Saos-2 cell apoptosis by inhibiting oxidative stress-mediated autophagy. However, there were still a notable limitation in our study. The effects of curcumin on the lipotoxicity of Saos-2 cell in our present study might not represent the actual condition of human diabetic osteoporosis. Therefore, this study is the first step toward assessing the use of curcumin as a bioagent for the treatment of diabetic osteoporosis. To this end, we intend to establish an animal model to further confirm our results with the same way.

## 5. Conclusions

This is the first study to report that curcumin protects against PA-induced human Saos-2 cell apoptosis via inhibiting oxidative stress and autophagy (Figure 7). Our result may offer new insights into the molecular mechanisms and treatment of lipotoxicity in diabetic osteoporosis.

## Data Availability

The data used to support the findings of this study are available from the corresponding author upon request.
